# Induced leg length inequality affects pelvis orientation during upright standing immediately following a sit-to-stand transfer: a pre-post measurement study

**DOI:** 10.1186/s12891-023-06302-3

**Published:** 2023-03-17

**Authors:** Simon P. Vella, Michael Swain, Aron Downie, Samuel J. Howarth, Martha Funabashi, Roger M. Engel

**Affiliations:** 1 Department of Chiropractic, Faculty of Medicine, Health and Human Sciences, Macquarie University, Sydney, NSW Australia; 2 Division of Research and Innovation, Canadian Memorial Chiropractic College, Toronto, ON Canada; 3 Department of Chiropractic, Université du Québec à Trois-Rivières, Trois-Rivières, QC Canada

**Keywords:** Pelvis orientation, Pelvis torsion, Low back pain, Functional movement, Sit-to-stand, Leg length inequality

## Abstract

**Background:**

Leg length inequality (LLI) greater than 20 mm has been associated with low back pain (LBP) and its correction is clinically recommended. Much less is known about the biomechanical effects that LLI below 15 mm has on pelvis orientation.

**Methods:**

Twenty-two adult participants (8 female) aged between 18 and 30 years without LBP were enrolled in the study and completed a series of sit-to-stand trials with no heel-lift (0 mm baseline) and heel-lifts of varying heights (5, 9 and 12 mm) placed in their right shoe. Three-dimensional kinematic data were obtained from the lower extremities, pelvis and thorax. Additional kinematic data were obtained from the left and right sides of the pelvis. The global orientation of the whole pelvis and relative orientation between the left and right sides of the pelvis were obtained in upright standing immediately upon completion of the sit-to-stand movement. Repeated measures ANOVAs were used to detect differences in sample means across the different levels of heel-lift (0, 5, 9, and 12 mm). The tests for within-subject effects determined overall significant differences between the means at the different levels of heel-lift induced LLI. Partial Eta-Squared was used to express the size for the main effect of heel-lift height. For each level of heel-lift, the estimated marginal mean and 95% confidence interval (95%CI) values of pelvis angles were illustrated graphically.

**Results:**

Left frontal plane rotation of the pelvis increased (p = 0.001), that is, the left side of the pelvis was lower than the right side of the pelvis, and anterior tilt of the pelvis decreased (p = 0.020) with a heel-lift height (applied on the right) as low as 5 mm. A significant main effect of heel-lift was only observed for the norm of rotations about all three axes for relative-pelvis orientation (p = 0.034). Post-hoc analyses did not reveal any statistically significant differences between the heel-lifts and the 0 mm baseline (p≥0.072).

**Conclusion:**

These findings suggest that correcting leg length inequality below the recommended threshold of 20 mm may influence pelvic orientation. Future work can investigate the effects of the altered orientations on spine loading and the clinical effects of corrections to minor leg length inequality.

**Supplementary Information:**

The online version contains supplementary material available at 10.1186/s12891-023-06302-3.

## Background

A significant amount of research has focused on the pelvis and its potential role in mechanical low back pain. A specific physical factor of interest to clinicians and researchers is the apparent asymmetry in pelvic orientation that may occur because of an inequality between leg lengths [1–3]. Specifically, leg length inequality (LLI) greater than 20 mm has been associated with scoliosis, pelvis asymmetry and increased mechanical work during gait and are typically seen in children and adolescents, as a result of developmental disorders, congenital disorders, lower limb fracture, hip dislocation or hip dysplasia [4–6]. Given its impacts, studies have been conducted to quantify biomechanical differences in pelvis orientation and neuromuscular function in the upright standing position either between people with and without LLI or by artificially inducing LLI in people with legs of similar length [7–10]. However, most of this work focuses on LLI that is at least 15 mm. This is due to most studies attempting to evaluate the amount of LLI required to change spinal parameters (e.g. lumbar, thoracic and cervical rotation) [11]. Much less research has investigated the biomechanical adaptations with either naturally occurring or artificially induced LLI that is smaller than 15 mm, despite suggestions that more than 90% of the population have been reported to have LLI less than 14 mm [12].

It is hypothesized that the pelvis, when forced down on the femoral heads in the presence of asymmetrical leg lengths, torsions as a result of asymmetrical loading and alters neuromuscular activity creating an imbalance in muscle tone and tension [3, 13–20]. This results in alterations in hip adduction and abduction motion and pelvis elevation [21]. Experimental studies have investigated these biophysical adaptations by using blocks, plates and shoe lifts to artificially induce LLI [1, 2, 11, 13, 22–29]. For example, studies have used custom-built sandals and simulation platforms to artificially induce LLI in participants to evaluate global pelvis orientation [1, 2, 22]. Their findings suggested that artificially induced LLI of at least 15 mm appears to increase global pelvic tilt and pelvic rotation during static upright standing.

Previous research evaluating changes in pelvis orientation during upright standing with induced LLI is limited by (1) the amount of time given to participants to adapt to the induced lift and (2) the instruction to participants to keep their legs as straight as possible. For example, a 10-week clinical trial that corrected LLI smaller than 10 mm in a group of participants with chronic low back pain demonstrated significant reductions in pain intensity (measured using visual analogue scale) and disability (measured by Roland Morris Disability Questionnaire) when compared to the untreated control group [30]. In another study, changes in pelvis orientation during upright standing where participants have been given adequate time (greater than 60s) to adapt to the heel lift have been demonstrated with induced LLI as low as 5 mm [2]. Thus, these studies likely are not capable of assessing the immediate effect of the induced LLI on pelvis orientation in upright standing under less constrained conditions. Understanding the immediate effect of LLI on pelvis orientation will provide new knowledge of biomechanical adaptations that occur with smaller LLI thresholds (< 15 mm) during upright standing, an area of research that remains unclear [11]. Performing a functional task such as the sit-to-stand (STS) movement immediately prior to measuring pelvis orientation in upright standing may the limit time subjects have to adjust to the heel lift and provide a better indication of the immediate biomechanical response to induced LLI.

Biomechanical studies often model the pelvis as a single rigid segment; however, it is anatomically comprised of multiple bony structures and synovial joints that permit intrapelvic movement between the bones. Thus, kinematics of the pelvis can be classified according to either whole or relative pelvis kinematics [31, 32]. Whole pelvis kinematics refers to the movement of the pelvic bones as one rigid structure, where global rotation of the whole pelvis occurs with respect to an external reference point. Relative pelvis kinematics are defined as intrapelvic movement i.e., movement of one side of the pelvis with respect to the other side [1]. An example of relative pelvis kinematics includes asymmetric pelvic motion whereby the upper ilium rotates in one direction while the contralateral lower pubis rotates in the opposite direction in a curvilinear path [33], pelvic torsion, which is frequently described as the lateral rotation of the pelvis in the frontal plane [31] and pelvic tilt in the sagittal plane [3]. Despite the claim of a dose-response relationship between LLI and the relative orientation between the innominate bones [34], accurate estimates for the magnitude and direction of pelvic torsion as a function of LLI have not been sufficiently established. Uncertainty remains around the level of association between LLI and pelvis kinematics, particularly pelvis orientation. Thus, the evaluation of asymmetric pelvic motion in the presence of LLI requires an investigation of relative pelvis kinematics [33].

The primary study objective was to evaluate the effect of induced LLI using heel-lifts of varying heights, up to 12 mm, on whole pelvic orientation in upright standing immediately following a STS movement. Consistent with previous research, we hypothesized that heel-lift induced LLI would result in altered whole pelvis orientation in the frontal plane during upright standing. Secondary objectives were to evaluate the feasibility of recording relative pelvis kinematics between the left and right side of the pelvis and the effect of heel-lift induced LLI on these measures.

## Methods

### Study design & setting

A single group before/after design was applied to study the effects of artificially induced LLI on pelvis orientation. The study was conducted at the Canadian Memorial Chiropractic College’s (CMCC’s) Human Performance Laboratory in Toronto, Canada between November 2019 and January 2020. Ethics approval was received from the CMCC Research Ethics Board (approval number: 1908B02). All participants signed a written informed consent prior to participating in the study.

### Participants

Volunteers responded to a public recruitment notice for the trial. To be included participants had to be between 18 and 30 years of age and have a body mass index less than 30 kg/m^2^. The upper age limit was chosen to minimise influence of degenerative and other age-related changes on the study findings. Potential participants were excluded if they had low back pain or lower extremity injury within 4 weeks of their scheduled data collection, history of fracture or osseous pathology of the spine or lower limbs, previous surgery to the spine or lower limbs, history of neurological or cardiovascular disease, history of cancer, currently pregnant, or taking medications that may have affected their balance or movement (e.g., tricyclic antidepressants, benzodiazepines [35]). Participants were also excluded if they had a structural [36] or functional [37] LLI greater than 15 mm (taken as the average of two measures between the medial malleoli of the tibia and the anterior superior iliac spine [38]), signs of pelvic torsion when assessed for symmetry in the upright position using palpation and visual estimation [39, 40], or scoliosis assessed using Adam’s test [41]. A practicing chiropractor with 8 years of experience assessed participants’ eligibility and conducted measurements of leg lengths. Leg length measurements were manually recorded by a research assistant.

#### Sample size estimate

A sample size of 22 participants was determined *a priori* based on a statistical power of 95% for a repeated measures analysis of variance (ANOVA), with a significance level of 5%, and assuming a medium effect size (F = 0.33; Partial Eta-Squared [h_p_^2^=0.1]) of each heel-lift on pelvis orientation in upright stance [42].

### Instrumentation

#### Kinetic

Three force plates monitored the forces and moments at the interfaces between the participant and their external environment to determine key points in the STS task (e.g., movement endpoint/upright standing). Two ground-mounted force plates (BP400600, AMTI Inc., Watertown, MA, USA) measured the reaction forces between the participant’s feet and the ground. The third force plate (OR6-7, AMTI Inc., Watertown, MA, USA) was mounted to a rigid support structure and used as the seat for all STS trials. Kinetic data were sampled at 2000 Hz and were synchronized with the kinematic data.

#### Kinematic

An optoelectronic motion capture system (Optotrak Certus, Northern Digital Inc., Waterloo, ON, Canada) was used to record pelvis kinematics in three-dimensions. Individual rigid plastic plates, each housing three infrared light emitting diodes (IREDs) were secured to the feet, shanks, pelvis (over 1st sacral tubercle) and thorax using a combination of Velcro® straps and tape. Two additional rigid plastic plates were secured to the left and right sides of the participant’s pelvis and overtop of an elastic belt that was wrapped around the participant’s body at the level of the iliac crests.

Digitised anatomical landmarks on the lower limb, pelvis, thorax, acromion and spine were used to aid the construction of segment-specific anatomical frames of reference during post-collection processing of kinematic data. Bilateral landmarks of the lower extremities were the; distal heads of the first and fifth metatarsals, medial and lateral malleoli, medial and lateral knee joint line and greater trochanter. Pelvic landmarks were the same as Howarth et al. [43]: including the left and right; iliac crest, anterior superior iliac spine and posterior superior iliac spine, and the 1st sacral tubercle and spinous process of the fifth lumbar vertebra. Thorax landmarks were the acromion processes (anterior aspect), the xiphoid process, the suprasternal notch and the spinous process of the twelfth thoracic vertebra. Each of the landmarks were referenced to the IREDs on the appropriate rigid plate and virtually monitored throughout data collection using mathematical rigid body transformations.

All kinematic data were expressed with respect to a righthand global coordinate system for the laboratory with its origin situated to the left of the participant and between the ground-mounted force plates and the force plate on the seat. The following convention was used for the global coordinate system: +X = forward, +Y = upward and + Z = right. Kinematic data.

were digitally sampled at 100 Hz.

### Protocol

Participants were instructed to wear tight-fitting garments and athletic footwear to their scheduled appointment. Demographic characteristics were collected for each participant after confirming their eligibility and obtaining informed consent. Prior to instrumentation, a research assistant measured the height of the participant’s knee joints in the standing position, which was then used to individualise the seat height, using half inch plywood boards, for the STS trials [43, 44]. The seat height was set at 110% of the participant’s standing knee joint height [44]. Once this position was determined, the participant was asked to adopt a comfortable stance by placing one foot on each of the ground-mounted force plates and to sit comfortably on the seat. Strips of tape were used to mark the most posterior margin of the buttocks and the placement each foot to standardise the participant’s starting position for each STS trial.

Kinematic instrumentation were then placed on participants followed by digitisation of the aforementioned anatomical landmarks. Each corner of the three force plates were also digitised in separate trials following instrumentation. A single 5-second trial was obtained with the participant standing in an anatomically neutral posture on the ground-mounted force plates.

Participants were then allowed to acclimate themselves to the instrumentation by practicing the STS movement. Participants were required to complete a minimum of one and maximum of three practice trials. Next, participants completed a single 10-second trial of marching on the spot as a check of the kinematic instrumentation followed by a baseline set of 3 STS trials without a heel-lift (i.e. 0 mm heel-lift condition). To study the effects of artificially induced LLI on pelvis orientation immediately upon completion of the STS movement, a series of heel-lifts (Anatomical Heel-Lifts, St. Ives, Australia) of pre-specified height (5 mm, 9 and 12 mm) were placed in the participant’s right shoe in either ascending or descending order of height. The order of heel-lift height for each participant was randomly determined based on an *a priori* pre-determined sequence. Participants were blinded to the height of each heel-lift. Kinematic and kinetic data were obtained from 3 STS trials with each heel-lift height. Thus, each participant completed a total of 12 experimental STS trials. A minimum of 30-seconds rest was provided between trials within a heel-lift condition and at least 60-seconds rest was provided between successive heel-lift conditions. During the rest periods, participants were instructed to move around but were restricted from sitting, stretching or performing vigorous activity.

Each STS trial began with the participant’s feet flat on the ground and buttocks on the seat according to the previously marked locations. Prior to each STS trial, participants were verbally instructed to: “Please stand up straight as quickly as you can. Begin by sitting up straight. Do not move your feet and keep your arms folded across your chest throughout the trial. Remain standing until the assistant indicates you can sit down.” An investigator observed each trial to avoid any issues that may have impeded performance of the STS trial (e.g., restriction of movement by pulling of cables) or acquisition of the kinematic data (e.g., marker obstruction). If the participant changed position, failed to keep their arms across their chest or interfered with the IREDs and/or their wiring, the test was considered a failure and the trial was recollected.

### Data processing and analysis

All kinematic and kinetic data were imported to Visual3D (C-Motion Inc., Germantown, MD, USA) and processed. Force plates were located within the laboratory using the coordinates of their digitised corners. This also allowed for force plate data to be expressed with respect to the laboratory’s global reference frame. Kinematic data of the first upright standing trial were used to create a participant-specific 8-segment linked rigid model of the lower extremities (including the feet, shanks, femurs), pelvis and thorax. Using the digitised anatomical landmarks, anatomical frames of reference were constructed for each segment and were used to define the neutral joint position for each joint. Two additional representations for the pelvis were developed to monitor relative kinematics between the left and right sides of the pelvis. The anatomical frames of reference for these additional representations were constructed to be coincident with that of the pelvic segment that was part of the previously described 8-segment linked rigid model. These additional pelvic segments facilitated the quantification of relative movement between the right and left sides of the pelvis during the experimental trials. Global orientations of the pelvis in each of the laboratory’s cardinal planes (sagittal, frontal, transverse) were represented by a time-varying three-dimensional vector of angles throughout the STS trials. Relative intrapelvic movements between the right and left sides of the pelvis were quantified throughout each STS trial using a joint coordinate system decomposition. A mediolateral-anteroposterior-axial decomposition sequence was used to determine both the global pelvic orientations and the left-right pelvic joint angles.

Instants for the initiation and termination of movement were identified using the kinematic time-series data as well as the ground reaction forces (kinetic data) from each STS trial [43]. Our outcome of interest (i.e., changes in pelvic orientation with heel-lift induced LLI) was in the upright stance and therefore measures were taken at the termination instant of the STS movement. The decision to evaluate overall and relative pelvic orientation with heel-lift induced LLI immediately following a complex motor task was to address the potential limitation of habituation in previous studies that assessed changes in pelvis orientation with induced LLI during static upright stance. The three-dimensional angles representing the global (whole) and relative pelvis orientations along with the Euclidean norm of these angles were extracted from each STS trial. The norm was intended to represent the overall deviation of the pelvis from either the global reference frame or between sides of the pelvis and was calculated by the equation N = SQRT(X^2^ + Y^2^ + Z^2^). An average of the extracted values across the three trials in each heel-lift condition were used as dependent measures in subsequent statistical analyses.

### Statistical analysis

Descriptive statistics were used to characterize the sample. Whole and relative pelvis orientations were summarized across the sample for each heel-lift height. Summaries of three-dimensional pelvis orientations were calculated for individual components and the norm of these components for both the whole pelvis and relative pelvis.

Repeated measures ANOVAs were used to detect differences in sample means across the different levels of heel-lift (0, 5, 9, and 12 mm). The tests for within-subject effects were used to determine if there were overall significant differences between the means at the different levels of heel-lift induced LLI, expressed as the F-statistic (degrees of freedom, error degrees of freedom) and p-value. The size for the main effect of heel-lift height was expressed using Partial Eta-Squared. For each level of heel-lift, the estimated marginal mean and 95% confidence interval (95%CI) values of pelvis angles were illustrated graphically. A series of *post-hoc* between-level analyses were conducted using Bonferroni correction that estimated the mean differences (95%CI) in pelvis angle of rotation between levels of heel-lift induced LLI (referenced to 0 mm) to represent the estimate of heel-lift effect at each level. All statistical analyses were conducted using IBM SPSS Statistics for Windows, Version 25.0 (Armonk, NY: IBM Corp). An alpha level of 0.05 was used for all statistical analyses.

## Results

### Participants

**Fig. 1:**
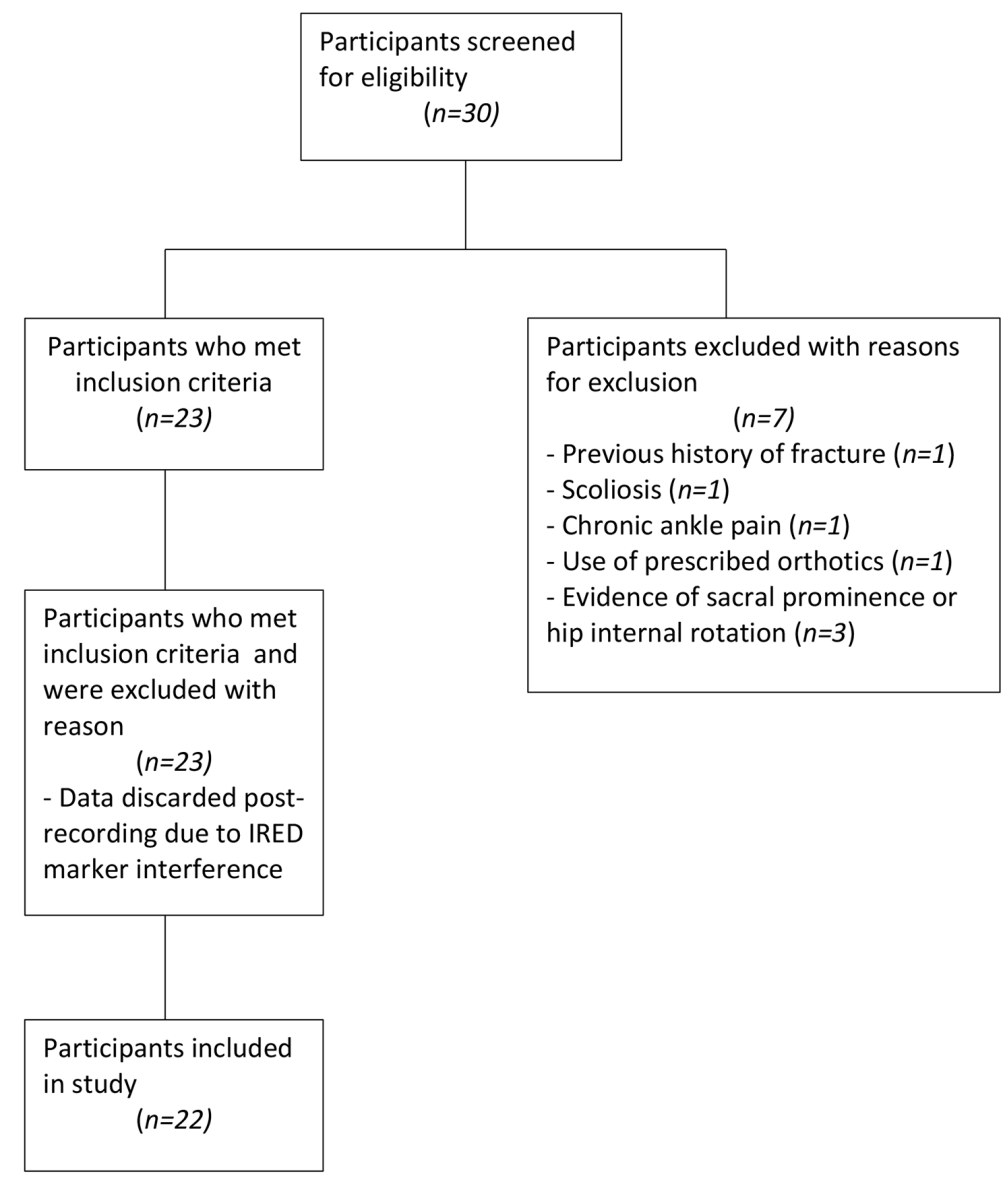
Participant flow diagram with reasons for exclusion

A total of 30 individuals were screened for eligibility for the trial. Twenty-two participants provided written consent and were enrolled in the study. Figure 1 shows participant flow and reasons for exclusion. Characteristics of included participants are reported in Table 1.

**Table 1:** Characteristics of participants. Values for age, height, mass and leg lengths are reported as means with standard deviations in parentheses

Total participants		N = 22 (36% female)
Age (years)		25.0 (1.6)
Height (m)		1.72 (0.10)
Mass (kg)		72.6 (12.3)
BMI > 25 (kg/m^2^)		N = 8
Participants categorised by leg length:	Equal leg length	N = 7 (32%)
	Shorter right leg	N = 4 (18%)
	Shorter left leg	N = 11 (50%)

### Heel-lift effect on pelvic orientation

Statistically significant main effects of heel-lift height were observed for orientations of the whole pelvis in the frontal (F(3, 63) = 53.466, p < 0.001, η_p_^2^ = 0.718) and sagittal (F(3, 63) = 9.590, p < 0.001, η_p_^2^ = 0.314) planes at the termination point of the STS movement (Table 2). Whole pelvis rotations increased with induced LLI (i.e. on the lifted side) in the frontal plane in 82% of participants while 5% of participants had no change, and in the sagittal plane, 77% of participants had an increase in posterior (i.e. tilting) rotations while 9% had no change in pelvis position. In comparison to the 0 mm reference condition, the heel-lifts increased the left lateral rotation (p≤0.001) and reduced the anterior tilt (p≤0.020) of the pelvis (Additional file 1). Overall there was no effect of the heel-lift height on the norm of the pelvis orientation with respect to the global reference frame at the end of the STS movement (F(3, 63) = 0.282, p = 0.839, η_p_^2^ = 0.013) (Table 2).

**Fig. 2:**
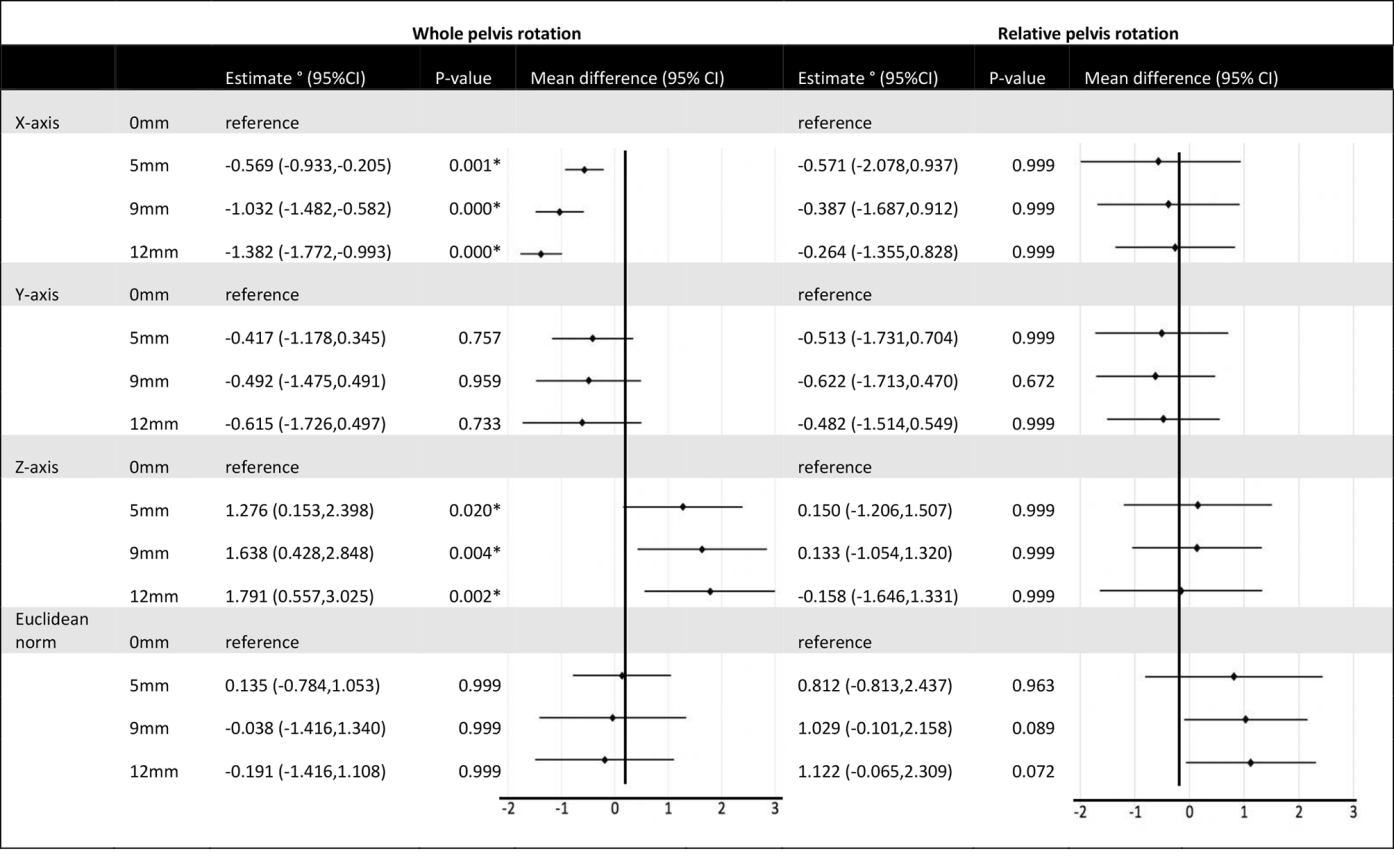
Mean difference (95%CI) and level of significance in pelvis angle of rotation with heel-lifts (5 mm, 9 and 12 mm) compared to reference (0 mm). * indicates the mean difference is significant at the 0.05 level. Interpretation of polarity with reference to 0 mm condition: x-axis: values below zero = towards left rotation, values above 0 = towards right rotation; z-axis: values below 0 = towards posterior tilt, values above zero = towards anterior tilt; y-axis: values below 0 = towards left axial rotation, values above 0 = towards right axial rotation

The only significant main effect for the relative orientation between the left and right sides of the pelvis at the termination point of the STS movement was for the norm of the three-dimensional relative orientation (F(3, 63) = 3.081, p = 0.034, η_p_^2^ = 0.128) (Table 2). There was an apparent increase in the magnitude of the relative orientation between the left and right sides of the pelvis with increased heel-lift height; however, this was not confirmed by the *post-hoc* comparisons between the 0 mm reference condition and any of the heel-lift heights (p≥0.072) (Fig. 2).

**Table 2:** Mean angle (standard deviation) of whole pelvis and relative pelvis orientation in upright standing for each heel-lift height

Whole pelvis rotation				Relative pelvis rotation			
0 mm	5 mm	9 mm	12 mm	0 mm	5 mm	9 mm	12 mm
**x-axis** (frontal plane)							
*0.16*	*-0.41*	*-0.87*	*-1.22*	-0.63	-1.20	-1.02	-0.90
*(1.89)*	*(2.00)*	*(2.02)*	*(2.02)*	(2.37)	(3.20)	(3.07)	(2.65)
**y-axis** (transverse plane)							
1.00	0.58	0.51	0.39	0.32	-0.19	-0.30	-0.16
(3.24)	(3.53)	(3.53)	(3.16)	(2.76)	(2.68)	(2.73)	(2.72)
**z-axis** (sagittal plane)							
*-1.99*	*-0.71*	*-0.35*	*-0.20*	0.32	0.47	0.46	0.16
*(4.21)*	*(4.32)*	*(4.37)*	*(4.03)*	(2.30)	(3.41)	(3.46)	(4.08)
**Euclidean norm**							
5.53	5.66	5.49	5.34	*3.92*	*4.73*	*4.95*	*5.04*
(2.49)	(2.02)	(2.56)	(1.95)	*(1.89)*	*(2.84)*	*(2.28)*	*(2.48)*

Estimates in degrees. Standard deviation in parentheses All values are reported as degrees. Statistically significant main effects of heel-lift height (p < 0.05) are denoted by italicized font. Interpretation of polarity with reference to 0 mm condition: x-axis: values below zero = towards left rotation, values above 0 = towards right rotation; z-axis: values below 0 = towards posterior tilt, values above zero = towards anterior tilt; y-axis: values below 0 = towards left axial rotation, values above 0 = towards right axial rotation

## Discussion

Asymmetry of pelvic orientation in the presence of LLI has been proposed as a contributing factor for the development of mechanical LBP [9]. The relationship between the extent of LLI and differences in pelvic orientation may provide a biomechanical foundation that links with LBP. Previous cross-sectional biomechanical studies investigating pelvis kinematics have been limited by the study methodology allowing participants to adapt to induced LLI which may confound the association between induced LLI and pelvis kinematics. Our biomechanical study on healthy people focused on evaluating the immediate effect of induced LLI on whole pelvis and relative pelvis orientation which limits confounding from compensation in the kinetic chain observed in previous studies. We found that heel-lift induced LLI as low as 5 mm altered whole pelvis rotations in 82% of participants in the frontal plane (i.e. pelvic hiking on the lifted side) and in 77% in the sagittal (i.e. posterior pelvic tilting) planes during upright stance at the end of a STS movement, although rotations are of small magnitude. Side-to-side differences in leg length also supposedly induce differences in orientation between the right and left sides of the pelvis. The overall difference in orientation between the left and right sides of the pelvis demonstrated a significant main effect of heel-lift induced LLI; however, post-hoc tests showed that none of the heel-lift heights were significantly different in the relative angle of pelvis rotation compared to no heel-lift.

### Clinical significance

Comparatively few studies focus on biomechanical adaptations to induced LLIs that are smaller than 15 mm. This may be because current clinical guidelines support a 20 mm LLI threshold for using insole heel-lifts and shoe-lifts in individuals with chronic back pain [45–47]. It is reported that LLI of 20 mm or greater is linked with an increased risk of LBP and hip pain [45, 46]. Interestingly, a randomized controlled study completed by Defrin et al. found that correction of LLI of 10 mm or less significantly reduced LBP intensity after a 10-week intervention in comparison to a control group of participants with LBP that did not receive the intervention [30]. Defrin et al. did not measure pelvic orientation as part of their study, which precludes an association between changes to pelvic orientation from the insole LLI correction and the intensity of LBP; however, the findings from our study indicate that changes to pelvic orientation in upright standing with induced LLI as low as 5 mm is consistent with previous work using rasterstereography, suggesting that a change in pelvic orientation may have been induced by the LLI correction in the previous clinical study [2]. Furthermore, Knutson et al. suggests that approximately 90% of the population have LLI that is less than 14 mm, yet the effect of heel-lift use below 12 mm in the clinical management of low back and pelvis pain remains unclear and an area that requires further research [9].

Unlike previous work [2, 3, 13], we did not observe any influence of heel-lift induced LLI on relative orientation between the left and right sides of the pelvis in upright standing. These studies collectively reported a posterior tilt of the pelvis on the lifted (i.e. longer) side that was countered by anterior tilting of the pelvis on the non-lifted (i.e. shorter) side. Methodological differences between previous investigations and ours may have contributed to the discrepancy in findings related to the relative pelvic orientation. Specifically, in our study, participant instrumentation and the measurement system used, as well as the chosen experimental task and instructions provided to participants were different than those used by previous studies. For example, previous studies have predominately used upright standing (static) or gait (dynamic) as the chosen task for evaluation [1, 2, 7, 22, 25, 26]. Those studies that used upright standing as the task will often allow participants to acclimate to the induced LLI and will also instruct participants to stand with both legs as straight as possible. The current investigation chose to evaluate the influence of heel-lift induced LLI on pelvic orientation in upright standing immediately following completion of STS movement. Furthermore, participants were not provided with specific instructions about the upright standing posture at the end of the STS movement. Both of these decisions were made to focus our evaluation on the acute effects of the heel-lift induced LLI on pelvic orientation in the upright standing posture.

## Limitations

A number of limitations need to be considered when interpreting the data from this study. While our data contributes to understanding the relationship between heel-lift induced LLI and pelvis orientation, our study is limited to healthy young participants. The findings may not be generalisable to clinical samples and should be interpreted with caution. Three-dimensional motion analysis accuracy is determined by factors such as the reliability of marker placement [48] While there was potential for movement of the rigid plastic markers during the STS phase in our study, procedures were implemented to reduce the risk of this occurring such as instructing participants to wear tight fitting garments and reinforcing marker placement with Velcro® straps, tape and the use of an elastic belt. It is possible that the recorded kinematics are susceptible to soft tissue artefacts and that marker inconsistency may have led to a degree of measurement error in our results. Future studies need to validate this approach. For example, cadaveric research using bone pins and surface-based markers. Alternatively, using a different approach altogether such as rasterstereography may be warranted to assess relative pelvis kinematics similar to Michalik et al. [49]. In designing this study, we sought to evaluate adaptations that occurred at the pelvis with induced LLI of at most 12 mm. This design decision precluded an analysis of postural adaptations to induced LLI that might have also occurred at the hips, knees and ankles. Additionally, the initial foot position may influence STS biomechanics at the ankle, hip and knee joints which is why foot position was controlled in our protocol. It is possible that the observed changes in whole pelvis orientation in response to the induced LLI were mediated, either positively or negatively, by concomitant adaptations in the lower extremities at the end of the sit-to-stand. Future research should expand on this study and evaluate (i) lower limb kinematic changes that are associated with LLI below 12 mm and (ii) the change this has on relative pelvis orientation. In addition, there was variation in how participants responded to heel-lifts, and the use of a heel lift in those participants with a shorter right leg may have corrected their LLI. This study was not powered to explore heterogeneity of response (e.g. by sub-grouping). Finally, experimental before/after study designs contain a level of selection bias due to the controlled selection of participants. By implementing strict eligibility criteria to include participants with presumably optimal pelvis mechanics, common afflictions such as low back and pelvis pain were excluded. We acknowledge this represents a trade-off between representativeness and generalisability of the findings.

## Conclusion

The findings of this study provide new knowledge to advance our understanding of the association between heel-lift induced LLI and pelvis orientation in asymptomatic people. Overall, the findings support an association between heel-lift induced LLI and whole pelvis frontal and sagittal plane rotations but raises doubt on an association with relative pelvic torsion. These findings may help inform healthcare practitioners who aim to understand pelvis mechanics in people with LLI. Future research should evaluate whether internal shoe heel-lift interventions provide clinical effects on pelvis kinematics in people seeking care for back and pelvis pain.

## Electronic supplementary material

Below is the link to the electronic supplementary material.

Supplementary Material 1

## Data Availability

The datasets used and/or analysed during the current study are available from the corresponding author on reasonable request.
