# Supplementary material for: Induced leg length inequality affects pelvis orientation during upright standing immediately following a sit-to-stand transfer: a pre-post measurement study
Source: BMC Musculoskelet Disord. 2023 Mar 17;24:203. doi: 10.1186/s12891-023-06302-3 (PMC10022171; doi:10.1186/s12891-023-06302-3)
Supplement: Supplementary file 1 — Supplementary Material 1 [file 12891_2023_6302_MOESM1_ESM.docx]

| **Whole pelvis rotations (°)** | **Relative pelvis rotations (°)** |
| --- | --- |
| a. | b. |
|  |  |
|  |  |
|  |  |

**Additional file 1.** Estimated marginal mean angle (95% CI) of (a) whole pelvis and (b) relative pelvis rotations (°) in X-, Y-, Z-axes and the Euclidean norm of rotation (shaded) at each level of heel-lift (0mm, 5mm, 9mm, 12mm). Interpretation of polarity with reference to 0mm condition: x-axis: values below zero = towards left rotation, values above 0 = towards right rotation; z-axis: values below 0 = towards posterior tilt, values above zero = towards anterior tilt; y-axis: values below 0 = towards left axial rotation, values above 0 = towards right axial rotation. For relative pelvis rotations, x-axis: values above 0 = right pelvis rotated to right with respect to left pelvis in frontal plane, values below zero = right pelvis rotated to left with respect to left pelvis in frontal plane; y-axis: values above zero = right pelvis rotated to left with respect to left pelvis in transverse plane, values below zero = right pelvis rotated to right with respect to left pelvis in transverse plane; z-axis: values above zero = right pelvis rotated posterior/backward with respect to left pelvis in sagittal plane, values below zero = right pelvis rotated anterior/forward with respect to left pelvis in sagittal plane
